# Paramagnetic Functionalization of Biocompatible Scaffolds for Biomedical Applications: A Perspective

**DOI:** 10.3390/bioengineering7040153

**Published:** 2020-11-28

**Authors:** Simona Bettini, Valentina Bonfrate, Ludovico Valli, Gabriele Giancane

**Affiliations:** 1 Department of Innovation Engineering, University Campus Ecotekne, University of Salento, Via per Monteroni, 73100 Lecce, Italy; simona.bettini@unisalento.it; 2 National Interuniversity Consortium of Materials Science and Technology, INSTM, Via G. Giusti, 9, 50121 Firenze, Italy; 3 Department of Cultural Heritage, University of Salento, via D. Birago, 64, 73100 Lecce, Italy; valentine.bonfrate@unisalento.it; 4 Department of Biological and Environmental Sciences and Technology (DiSTeBA), University Campus Ecotekne, University of Salento, Via per Monteroni, 73100 Lecce, Italy

**Keywords:** magnetic nanoparticles, hydrogel, scaffold, tissue engineering, hard tissue, soft tissue

## Abstract

The burst of research papers focused on the tissue engineering and regeneration recorded in the last years is justified by the increased skills in the synthesis of nanostructures able to confer peculiar biological and mechanical features to the matrix where they are dispersed. Inorganic, organic and hybrid nanostructures are proposed in the literature depending on the characteristic that has to be tuned and on the effect that has to be induced. In the field of the inorganic nanoparticles used for decorating the bio-scaffolds, the most recent contributions about the paramagnetic and superparamagnetic nanoparticles use was evaluated in the present contribution. The intrinsic properties of the paramagnetic nanoparticles, the possibility to be triggered by the simple application of an external magnetic field, their biocompatibility and the easiness of the synthetic procedures for obtaining them proposed these nanostructures as ideal candidates for positively enhancing the tissue regeneration. Herein, we divided the discussion into two macro-topics: the use of magnetic nanoparticles in scaffolds used for hard tissue engineering for soft tissue regeneration.

## 1. Introduction

The use of nanoparticles (NPs) with different shapes, dimensions and functionalizations has come to play a significant role in the so-called theranostic applications [1,2] and more in general in the fabrication of bio-medical devices [3,4,5,6]. The intrinsic nature of the nanoparticles plays, of course, a central role in the choice of the particular application field. For example, ZnO and silver nanoparticles are used for their antibacterial properties [7,8]; gold nanoparticles have been demonstrated to be excellent photothermal agents [9,10,11] able to produce a temperature increase as a consequence of luminous photon absorption; upconverting NPs have been applied for ROS generation for cancer cells treatment [12,13,14]. In this context, the use of the magneto-responsive NPs deserves a special attention. In particular, iron oxide nanoparticles were used in a plethora of bio-medical applications. Fe_x_O_y_ and doped-Fe_x_O_y_ [15] nanostructures were employed as contrast agents in MRI [16,17], for promoting hyperthermia under the action of an external magnetic field [18,19], for magnetic targeted drug delivery [20,21] and tissue engineering [22]. In particular, tissue engineering is focused on the realization of 3D scaffolds for tissue repair, regeneration and cell cultures. Anyway, the realization of an in vivo scaffold or, even more complex, an organ appears very challenging. With this aim, the bio-magnetic approach based on the generation of a mechanic stimulus as a consequence of a magnetic field application shows a huge potential [22]. Several strategies based on magneto-responsive bio-based films [23,24,25], hydrogels [26,27], fibers [28] and 3D scaffolds [29,30,31] were used for disease treatment and repair of bone [32], muscle [33] and nerve tissues [34], by means of external magnetic stimulus. 

It is mandatory, of course, that the magnetic responsive nanoparticles (MNPs) have to be safe and biocompatible [35]. For this reason, the most used nanoparticles for biomedical magnetic stimulated applications are mainly, but not exclusively, represented by the iron-based nanoparticles [36]. Furthermore, the recent advances in the NPs’ synthetic procedures allow us to modulate the nanoparticles’ dimensions, shape and responses to the magnetic field. 

Before starting to examine the different approaches used to obtain magneto-responsive bio-compatible scaffolds, a quick introduction to the most important magnetic properties of the matter will be reported herein. 

Magnetic field intensity, usually reported as *H*, is correlated to the magnetic field *B* in the vacuum by the simple relation:

(1) $$B=\mu_{0}H$$

where *μ*_0_ is the vacuum permeability [37].

In presence of a magnetic field, the magnetic momentum of some materials can be aligned according to the magnetic field lines and this phenomenon will induce an enhancement of the magnetic field. Then, Equation (1) will be rewritten as follows:

(2) $$B=\;\mu_{0}H+\mu_{0}M$$

where *M* is called magnetization. 

*M* is directly proportional to the magnetic field *H*:

(3) $$M=\chi_{m}H$$

*χ_m_* is the magnetic susceptibility. 

Equation (3) allows us to express the magnetic flux density as a function of only *H* not only in vacuum:

(4) $$B=\mu_{0}H+\mu_{0}M=\mu_{0}\left(H+\chi_{m}H\right)=\mu_{0}\left(1+\chi_{m}\right)H=\mu H$$

where *μ* is the magnetic permeability of the solid [38]. 

In a diamagnetic solid, a weak and not-permanent magnetism is observed in the presence of an external magnetic field. 

The atoms of a paramagnetic material have permanent dipolar moments that, in the absence of an external magnetic field, are casually arranged giving a macroscopic magnetization equal to zero. When an external magnetic field is applied to a paramagnetic material, it will induce the alignment of the atomic dipoles with an amplification of an external magnetic field and *χ_m_* > 1.

Superparamagnetism is a feature of some nanoparticles: if the nanostructures are as small as a magnetic domain an external magnetic field applied to the NPs can magnetize them but the magnetic susceptibility appears higher than the corresponding *χ_m_* of the bulk material. When the external magnetic stimulus is removed, the paramagnetic substances will not preserve the magnetically imposed order. 

Excellent candidates for the preparation of magneto-responsive materials appear to be iron and iron oxide NPs. Iron-based nanostructures are not the only magneto-responsive materials used for the synthesis of smart materials. In fact gadolinium and manganese-based NPs have been reported in the literature for applications in different fields [39,40,41,42], even though the most used are the iron-based nanostructures. Multiple advantages in the use of iron oxide NPs if compared with others para and superparamagnetic nanostructures (MNPs) have been observed [43]. First of all, the synthetic procedures are usually easy, economic, environmental friendly and healthy [44,45]; furthermore, it is easy to modulate the chemical physical features of the NPs [25]. Again, even though the fate of the iron oxide NPs used for bio-applications in vivo and in vitro is still debated [36,46,47,48], they are usually considered safe and the possibility to functionalize the nanoparticles’ surface by means of organic and inorganic compounds makes them very versatile for several types of bio-applications [18,30,49,50].

In the present perspective, an overview of recent examples of scaffolds decorated with iron-oxide-based NPs used for tissue regeneration will be presented. In particular, we will consider the role of the paramagnetic and superparamagnetic iron-based nanostructures in the regeneration of hard and soft biological tissues promoted by the action of an external trigger or by the peculiar features of the NPs used to functionalize the biological device. Of course, scaffolds obtained with different materials and manufactured by different procedures are herein reported; it is worth underlining that all of them, regardless of the tissue type, in general, need to be biocompatible and biodegradable and to have an internal porous structure balanced with specific mechanical properties depending on the kind of tissue to be repaired [51]. The balance between porous architecture and mechanical features is fundamental to allow cell infiltration and attachment as well as local vascularization [52]. Often, in the case of hard tissue regeneration, a 3D structure to drive cell differentiation is required, whilst injectable hydrogels appear more suitable for soft tissue regeneration.

## 2. Magneto-Responsive Scaffolds for Hard Tissue Regeneration

In biology and medicine, hard tissue is identified as a tissue composed by cells inserted in a dense or mineralized extra cellular matrix that gives rigidity and/or elasticity to the tissue. In the animals, hard tissues are bone, dentine or tooth enamel.

The cartilage tissue deserves a separate discussion, and it cannot be classified as hard tissue. Both the mechanical and biological features of cartilage tissue are deeply different from the bones or tooth enamel, even though they can be considered strictly connected. Both cartilage and bone tissues derive from the same osteoprogenitor cells and the derived cells (osteoblast and chondroblast) secrete extracellular material where they undergo maturation. At last, both are connective tissues devoted to structural support. For all these reasons, magnetically triggering scaffolds for cartilage tissue repair and regeneration will be reported in the present section within bone and dental tissues. 

The use of MNPs nanoparticles in the fabrication of scaffolds for the hard tissue regeneration are justified at least by three different reasons: first, dispersed nanoparticles, usually iron oxide nanoparticles, but not exclusively [53], often show a positive effects on the cell adhesion, viability and proliferation [54]; second, the possibility to promote peculiar geometry, such as, for example, the preferential pathway for cell growth, is easily achieved by using an external magnetic field [55]; third, the dispersed nanoparticles entrapped in the scaffold matrix can be stimulated by a static or alternated external magnetic field (SEMF and AEMF, respectively). In this context, the magnetic field can be imposed to influence the scaffold fabrication, applied during the cell growth or it can be applied at all. 

### 2.1. MNPs Enriched Scaffolds without the Application of an External Magnetic Stimulus

The presence of nanoparticles can influence the mechanical properties of the matrix where they are embedded [56,57], its chemical features [58] and, above all, the cell adhesion and the proliferation [59]. For example, Wu and collaborators demonstrated that the presence of ad hoc synthesized superparamagnetic Fe_3_O_4_ [60] nanoparticles dispersed in hydroxyapatite (HA) scaffolds enhanced the cellular proliferation [61,62]. In fact, when scaffolds were immersed in a biologic medium, they interacted with the macromolecules, particularly with proteins, resulting in the formation of the so-called protein corona on their surface. The hydroxyapatite scaffold, prepared by the authors, simulated the inorganic part of the bone tissue and the presence of the iron oxide nanoparticles clearly influenced the protein corona composition. It increased the G-protein coupled receptors and MAPK/ERK pathway that promoted the cascade of signals and then the proliferation of the MC3T3-E1 cell cultures. The protein corona alteration, as a consequence of the simple presence of the superparamagnetic nanoparticles, was evidenced both in vitro and in vivo experiments. As proposed by the authors, the biological differences, particularly on the cell proliferations, observed comparing the HA and the iron oxide MNPs-decorated hydroxyapatite scaffolds lies in the influence exerted by the magnetic domains in the nanoscale field. In fact, it was demonstrated that the magnetic field plays an important role in the formation of the protein corona [63], then the magnetic properties of the MNPs are crucial for tuning the cellular uptake. 

The role of the magnetic field provided from the nanodomain of paramagnetic nanoparticles embedded in the HA scaffolds was pointed out onto both the osteoblasts and osteoclasts [64]. It was demonstrated that the presence of the paramagnetic nanoparticles in the hydroxyapatite scaffold induced the osteoblast growth and proliferation as a consequence of the modification of the osteoclast-derived exosomal cargo and of the reduction of the exosome uptake efficiency by osteoblasts. The positive effects induced by iron oxide superparamagnetic nanoparticles dispersed in the HA scaffolds both in vivo and in vitro experiments were even reported when the Fe_3_O_4_ nanoparticles were co-dispersed in 3D HA matrix in the presence of alendronic acid (AA) [65], which is known to inhibit the osteoclast differentiation and to enhance the osteoblastic activity [66]. Further, the nanoparticles were capped by poly(ethylene glycol) functionalized with carboxyl groups in order to interact with calcium ion of HA. The simultaneous presence of the paramagnetic nanoparticles and the AA allowed to confer to HA scaffolds capability to both stimulate osteogenesis and to reduce osteoclast functions. The application of the proposed scaffold on femoral defect model of osteoporetic rats unequivocally showed that the simultaneous presence of AA and NPs strongly increase the bone repair capacity. Alendronic acid is just one example of the several bio-polymers and bio-compounds mixed in the presence of HA and MNPs. For example, a poly(lactic-co-glycolic acid) derivative (PLGA), a bio-compatible copolymer widely used in the bone tissue engineering [67], functionalized by means of carboxyl groups able to interact with HA moiety, and ad hoc obtained superparamagnetic iron oxide (Fe_3_O_4_) nanoparticles were proposed [68]. MNPs were obtained by means of the co-precipitation method and oleic acid, oleylamine and hexadecanol were used as capping agents and surfactant [67]. The adopted procedure to obtain the paramagnetic HA-based scaffold is very simple: the nanoparticles were directly added into the slurry and different aliquots of NaCl as a porogenous agent were simultaneously dispersed (scaffolds with 150–200 nm large pores were obtained). Then, NaCl was removed after the slurry formation simply by immersion in distilled water for 24 h. The mechanical properties and biocompatibility of the bare HA-PLGA and HA-PLGA decorated with MNPs were comparable and it is worth observing that bone repair in vivo activity and cell adhesion appeared slightly improved in the presence of MNPs. Finally, the application of an alternate magnetic field (500 KHz with an intensity of 3 mT) promoted hyperthermia for the presence of MNPs, even though, as reported by the authors, this feature was not tested neither for in vitro nor for in vivo experiments. 

Particular attention has to be paid to the use chitosan (CS) in tissue engineering [69] and in self-healing [70] magnetic triggered supports [71]. In fact, the cytocompatibility and biodegradability of chitosan even when mixed with gold [72], silver [73] and iron oxide nanoparticles proposed this natural polymer as a suitable candidate for tissue engineering. CS is largely used in a mixture with several materials [74], including hydroxyapatite [75]. For example, it is possible to promote an in situ precipitation procedure to obtain very uniform and homogenous chitosan/hydroxyapatite and chitosan/hydroxyapatite/MNPs scaffolds using genipin with the double role of biocompatible material for improving the osteointegration and as a crosslinker agent [76]. Cell viability studies demonstrated that, as already discussed, the presence of the iron oxide paramagnetic nanoparticles increased the cell viability and enhanced osteoblast maintenance and differentiation. Nano-hydroxyapatite (n-HA) and magnetite nanoparticles were directly obtained by in situ crystallization into chitosan/collagen matrices [77]. n-HA and Fe_3_O_4_ precursors were incorporated into the chitosan/collagen matrix [78,79] and the scaffold properties were investigated. The porosity, a crucial characteristic for the bio-supports, of the CS/Collagen/Fe_3_O_4_/n-HA scaffold appeared higher than the scaffold without the paramagnetic MNPs as well as the temporal stability. The mechanical properties of the composite containing the MNPs were significantly increased by the presence of the nanoparticles in the matrix (the compressive modulus of the CS/collagen/Fe_3_O_4_/n-HA scaffold was measured 2.515 MPa against the 2.150 MPa in the case of the bare CS/collagen/n-HA support). Osteoblast MC3T3-E1 cells were used to evaluate the cytoactivity and biocompatibility of the two scaffolds that were found to be very high; furthermore, the cells’ proliferation in the CS/collagen/Fe_3_O_4_/n-HA was higher than that recorded for the CS/collagen/n-HA scaffold: in the absence of nanoparticles, both in vivo and in vitro, osteoblast proliferation is merely observed on the external edges of the scaffold; on the contrary, the hierarchical nanostructure obtained when MNPs are present in the matrix improved the nutrients and osteoblasts migration throughout the scaffold. 

CS was mixed with micrometric Fe_3_O_4_ powders [80] in order to obtain magneto-responsive scaffolds for potential bone tissue regeneration without any crosslinker, by using the freeze-drying method [81]. The amount of microparticles used appeared crucial both for preserving the three-dimensional structure of the scaffold and for ensuring a high grade of crosslinking. Furthermore, even though any chemical cross-linker molecules were not used, the CS-based supports appeared very stable in physiologic condition up to 4 weeks. No decrease in the C2C12 cell viability was observed in vitro cultures confirming the possible use of this simple method for CS paramagnetic scaffolds preparation. 

CS matrix was enriched by Fe_3_O_4_ nanoparticles and GdPO_4_ nanorods for tumour photothermal therapy and bone tissue regeneration [82]. GdPO_4_ nanorods bioactive features were previously investigated and their angiogenesis and osteogenesis for bone regeneration were reported [83]. In fact, Gd^3+^ ions released from the scaffold GdPO_4_/CS/Fe_3_O_4_ induces the macrophage M2 polarization. In more detail, GdPO_4_ and Fe_3_O_4_ obtained by means of hydrothermal and co-precipitation methods were suspended in CS acetic acid (2%) solution. The suspension was frozen at −20 °C for 12 h and then it was freeze dried at −60 °C for 48 h. The obtained scaffolds appeared formed by a micro-pore structure that was preserved in the presence and absence of nanorods and of Fe_3_O_4_ nanoparticles. When the GdPO_4_/CS/Fe_3_O_4_ scaffold was placed in water at 37 °C for 24 h, a quick release of Gd^3+^ ions was observed, on the other side Fe^3+^/Fe^2+^ release appeared slower. As a consequence of Gd ion release, the cell proliferation is promoted both in vitro and in vivo; furthermore, the osteogenic mechanism was enhanced since Gd^3+^ activated the BMP-2/Smad/RUNX2 pathway, leading to superior osteoconductivity. The growth rate of bone tissue in vivo was measured to be three times higher in the case of the GdPO_4_/CS/Fe_3_O_4_ scaffold if compared to the bare CS scaffold. The role of Fe_3_O_4_ nanoparticles, of about 20 nm size, was reported to be both to improve the osteogenic activity and to promote photothermal therapy under NIR irradiation. In fact, when the scaffolds containing the iron oxide nanoparticles were implanted for in vivo experiments, bone metastasis was successfully treated since a temperature increasing up to 45.5 °C in a few minutes was obtained under NIR irradiation, inducing a significant tumour cell apoptosis. 

CS was even used to dissolve the calcium phosphate cement (CPC) in the presence of superparamagnetic γ-F_2_O_3_ nanoparticles [84]. Solid scaffolds were obtained dissolving CPC powder in 5% water solution of chitosan malate with a mass ratio of 2:1. MNPs were added to the solution both in powder and suspended in liquid phase. As highlighted by the authors, the best scaffolds were obtained when MNPs powder was used. Different amounts of MNPs were dispersed in the CS/CPC powder and then dispersed in water. 0%, 1%, 3% and 6% of superparamagnetic nanoparticles were used. Mechanical tests, performed after the scaffolds’ immersion in water for 24 h evidenced that the CS/CPC scaffolds enriched with MNPs showed improved mechanical properties. In fact, mechanical strength for the samples containing the nanoparticles was twice higher than the CS/CPC scaffold. Furthermore, the chemical physical characteristics of the MNPs increased the wetting ability of the scaffolds and, as a consequence, the protein absorption. It has to be reported that a high amount of MNPs was detrimental to cell spreading and bioactivity, probably as a consequence of cells ROS production in the presence of high iron oxide nanoparticles’ concentration [85,86]. On the other side, cell spreading and bioactivity appeared enhanced in the case of 3% containing CS/CPC/MNPs scaffold as well as very interesting results were obtained when alkaline phosphatase (ALP) activity, osteogenic expression and bone mineral synthesis were monitored for the scaffolds containing 1% and 3% of paramagnetic nanoparticles (twice higher if compared to the CS/CPC scaffold). 

**Figure 1:**
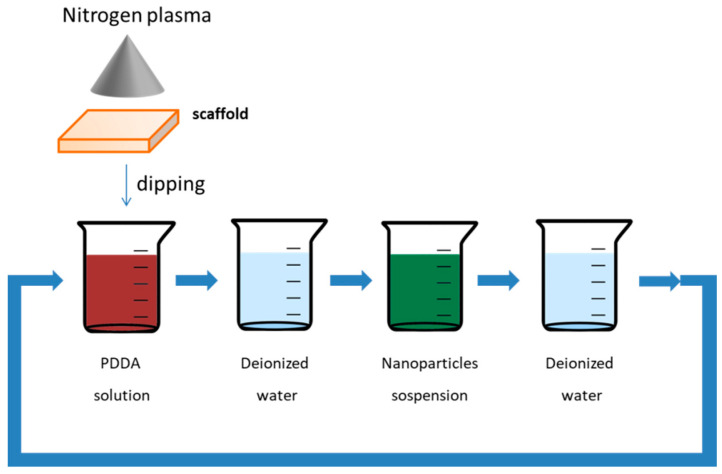
Schematic representation of the procedure used to fabricate the paramagnetic scaffolds [88].

The other family of chemical compounds largely used to obtain scaffold for tissue engineering is represented by the organic biocompatible polymers [87]. This class of compounds appears particularly appealing since they can be processed by means of many techniques, particularly by the use of 3D printing, a quick and economic technique used to fabricate three-dimensional scaffolds of different size and shape [12]. A mixture of poly(lactic-*co*-glycol acid)/polycaprolactone (PLGA/PCL) was used for realizing bio-compatible scaffolds decorated with both Fe_3_O_4_ and gold nanoparticles (AuNPs) [88] by means of 3D printing and layer by layer technique [89]. PLGA and PCL were dissolved in the presence of gelatine in 2,2,2-trifluoroethanol with glacial acetic acid. Then, the obtained solutions were extruded by means of the electrospun method and the 3D scaffolds were obtained. In a second step, the PLGA/PCL scaffolds were treated by means of nitrogen plasma for 30 s, 220 V and 1.5–1.8 A. This procedure allowed us to negatively charge the scaffold surface; then, the support was immersed in 2 mg/mL of poly-(diallyldimethylammonium chloride) [90] for 30 min and rinsed in water conferring a positive electrostatic charge to the scaffold. At this point, the support was immersed in the negatively charged nanoparticles suspension [91]. The scaffold was then freeze dried promoting the nanoparticles’ adhesion onto the surface. The procedure was repeated to increase the nanoparticles numbers into the scaffold (see Figure 1). The elastic modulus was enhanced both in the case of MNPs and AuNPs. Furthermore, the hydrophilic nature of the nanoparticles induced an enhancement of the scaffold wettability and the increased surface roughness improved the cell adhesion. Finally, as already highlighted, the γ-Fe_2_O_3_ magnetic features strongly enhanced the osteogenic differentiation of adipose-derive stem cells. 

**Figure 2:**
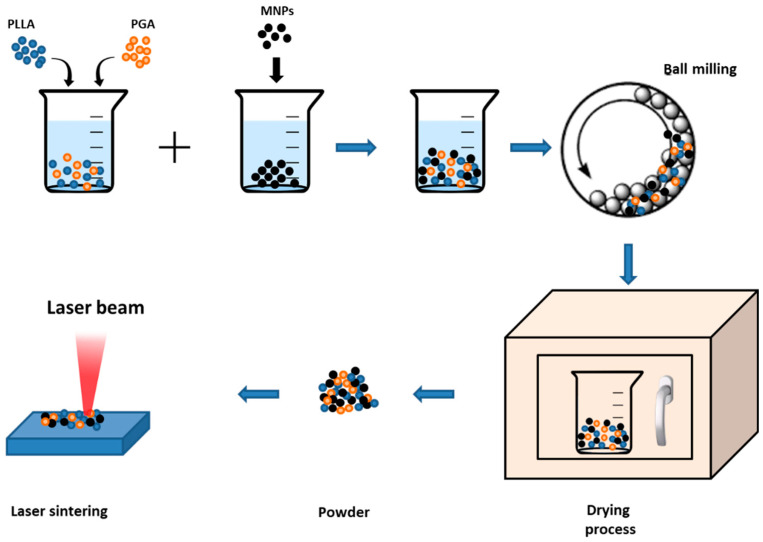
Schematic representation of the procedure used to obtain the scaffolds in [92]. A powder of well-dispersed poly-l-lactide (PLLA)-polyglycolic acid (PGA) and magnetic responsive nanoparticles (MNPs) mixture was synthetized, melted by means of a laser and then solidified to obtain the paramagnetic scaffold.

Another technique was used to build magneto-responsive scaffolds using organic polymers as proposed by Shuai and collaborators [92]. In Figure 2, the used procedure is schematized: poly-l-lactide (PLLA) [93,94] and polyglycolic acid (PGA) [95] powders were suspended in ethanol (1:1) and placed in an ultrasonic bath for 30 min. Then, an MNPs suspension (commercial 20 nm Fe_3_O_4_ nanoparticles) was added to the PLLA/PGA solution in different amounts (0, 2.5, 5, 7.5, 10 wt%) and next stirred by a grinding mill at 300 rpm/min for 120 min. The composite mixture was dried at 50 °C for 24 h and the obtained powder, composed by PLLA, PGA and MNPs, was irradiated by a laser with 2.5 W power, at 100 mm/s speed and 1 mm distance between the source and the target. The irradiating beam melted the powder and, when moved on another point, the composite material solidified and the paramagnetic scaffold was obtained with a porous 3D structure. 

As already discussed for reference [88], the MNPs increase the hydrophilic features of the scaffold containing the Fe_3_O_4_ nanoparticles and, for the scaffolds containing 0, 2.5, 5, 7.5 wt% iron oxide nanoparticles, a substantial improvement of the compressive strength and compressive modulus was observed. In vitro experiments clearly showed the positive influence of the superparamagnetic nanoparticles on cells adhesion (MG63 cell line was used) and, after 7 days of incubation, the cells appeared better spread on the scaffold surface (PLLA/PGA with MNPs 7.5%) and more numerous if compared to the bare PLLA/PGA scaffold. Furthermore, APL activity was used as early cell differentiation indicator. The highest APL was observed for the PLLA/PGA scaffold containing 7.5% MNPs as a consequence of the magnetic field of the MNPs at the nanoscale. So, the in vitro experiments suggested that the NPs’ incorporation enhanced the viability, proliferation and cell differentiation. Three-dimensional scaffolds were implanted to test their efficacy on rabbit radius bone defect and in vivo evidence confirmed the paramagnetic scaffolds’ ability to accelerate the bone regeneration. 

The versatility of biocompatible organic polymers allows one to mix them even with carbon nanotubes (CNTs). CNTs are largely used in several applications, for example, sensors [96], structural composite materials fabrication [97], electronic devices [98], solar cells [99] and biological and biomedical research [100]. In particular, commercial hydroxylated multiwalled carbon nanotubes (hCNTs) were used to form a hybrid adduct with iron oxide nanoparticles in poly(ε-caprolactone) scaffolds [101]. MNPs were synthetized by means of the co-precipitation method in the presence of CNTs. The adopted procedure allowed us to obtain a CNTs/MNPs hybrid compound as highlighted by transmission electron microscopy: 100 µm CNTs decorated with 20 nm iron oxide nanoparticles were clearly observed. The authors paid particular attention to the toxicity of the formed adduct; in fact, they tested the biocompatibility of the dyad CNTs/MNPs by an MTS test of mitochondrial activity, which is a well-known method used for measuring the cell metabolic activity of an SAOS-2 culture. The toxicity depended on the CNT/MNP concentration and it appeared not negligible. PCL scaffolds were obtained dissolving the polymer in dichloromethane and, after 24 h of vigorous stirring, the adduct CNTs/MNPs were added. A porous 3D matrix was obtained both in absence and in the presence of CNTs/MNPs that did not particularly influenced the three-dimensional morphology of the scaffolds. It is worth observing that the positive effects of the presence of CNTs/MNPs on the metabolic activity and on the adhesion of the seeded cells appeared limited by the toxicity of the hybrid dyad. 

As examples of hydrogel scaffolds, here, we report two recent contributes of injectable paramagnetic scaffolds for cartilage tissue regeneration. Hydrogel will be more deeply examined as tuneable scaffolds using an electromagnetic field as an external trigger. The viscosity and injectability of hydrogel scaffolds allow to use them mainly for cartilage tissue, even though they are used as platform for tissue regeneration and for drug delivery as well. Polyvinyl alcohol (PVA) and collagen were used to obtain the scaffold that was then enriched by Fe_3_O_4_ superparamagnetic nanoparticles [102]. Different volume ratio of PVA and collagen in water solution were used to obtain the scaffold and the nanoparticles were added drop by drop. Then, the mixtures were placed at −20 °C for 16 h and then kept at room temperature to melt them. It was observed that the mixture PVA/collagene, 96/5 showed the best mechanical features and a porosity structure with pores diameter of 20–50 µm and 150–300 µm. The MNPs’ presence did not influence the porosity of the matrix but improved the mechanical properties such as the Young modulus. The presence of iron oxide nanoparticles for the scaffold PVA/collagene,96/5 enriched with 5 mg Fe_3_O_4_ nanoparticles showed the best bioactivity when raw bone marrow mesenchymal stem cells were seeded. After seven days, cell stacking growth, cell proliferation and cell activity appeared higher than all the other Fe_3_O_4_/PVA/collagene,x/96/5 (x = 0, 2.5, 7.5, 10 mg). Similar results, both from a mechanical and from a biological point of view were obtained when iron oxide superparamagnetic nanoparticles were encapsulated in fibrin-agarose matrices [103], further testifying the huge potentialities of the use of MNPs in scaffolds for bone and cartilage tissue regeneration even in the absence of an external magnetic field. 

### 2.2. MNPs Enriched Scaffolds with the Application of External Magnetic Stimulus during the Scaffold Formation

The beneficial effects of the paramagnetic nanoparticles on the mechanical and biological properties of the scaffolds for the bone tissue regeneration can be further improved when appropriate external magnetic fields are applied to the obtained 3D structures. Intense, weak, static, low and high-frequency magnetic fields are used to modulate and to influence the bio-chemical responses of the biological materials seeded and grown on the scaffolds [104]. An electromagnetic field represents a very interesting external trigger for its low potential danger and the easiness of magnetic field generation and application [25]. For example, the simple application of a magnetic field produced by a permanent magnet was used to influence the macrophage polarization and to stimulate the osteogenesis of osteoblasts and the angiogenesis of endothelial cells seeded in a n-HA/poly-lactide scaffold enriched with 10 nm diameter spherical γ–Fe_2_O_3_ nanoparticles [105]. The SEMF intensity (from 5 to 10 mT) was obtained simply by reducing and increasing the distance among a neodymium magnet and the cell cultures (murine monocyte macrophage cell line), and the magnetic saturation was modulated by changing the nanoparticle concentration in the scaffold (from 0.2 and 2 emu·g^−1^). This simple procedure was enough to produce mechanical stress on the polymeric fibers as a consequence of the influence of H field on the MNPs aggregates and, thence, on the interface among the scaffold and cells membrane. The authors evidenced that this mechanical interaction suppressed the activation of membrane receptors TLR2/4, resulting in the inhibition of the production of IL1β, TNF-α and MCP-1 as well as the osteoclast differentiation cytokine MMP-9 and TRAP: mechanical forces generated by the applied magnetic field influenced the macrophage polarization grown in the scaffold enhancing the osteogenesis and the angiogenesis of epithelial cells. 

The application of AEMF was used for increasing the temperature in physiological medium by means of the phenomenon known as hyperthermia [106]: the γ-Fe_2_O_3_ MNPs were embedded in an n-HA matrix and the temperature increased up to 45 °C in 10 min (in the case of scaffolds with saturation magnetization of 12 emu·g^−1^). In this case, besides the positive effect on the bio-activity and mechanical benefits, an anti-cancer therapeutic application of the magneto-responsive scaffold can be proposed. 

The effect of an external moving magnetic field, applied simply by using a neodymium magnet, was evaluated on MC3T3-E1 cell cultures when superparamagnetic Fe_3_O_4_ and nano-hydroxyapatite were simultaneously dissolved in methoxy(polyethylene glycol)-polyalanine for obtaining an injectable hydrogel [107]. The composite approach, that is, the internal and external magnetism, appeared particularly interesting. In fact, without any application of an external field, the presence of the MNPs enhanced the cell viability and proliferation. As observed by the authors, the cell viability increased with the MNPs’ concentration: after 7 days, the cell viability appeared higher for the scaffold obtained using the highest nanoparticle concentration (1 mg mL^−1^). This can be imputable, as previously reported, to the magnetic field provided from the nanodomain of dispersed paramagnetic nanoparticles. Furthermore, the moving magnetic field positively enhanced the osteoblast differentiation.

**Figure 3:**
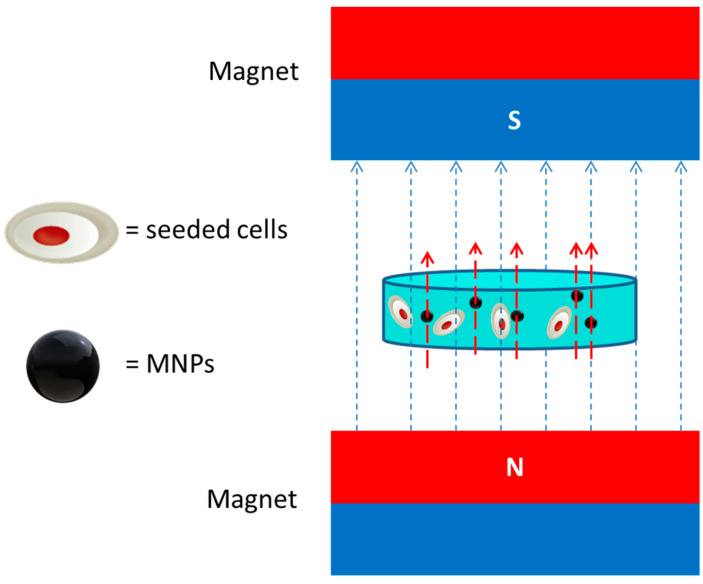
According to the reference [108], the amplification of the external magnetic field obtained by the presence of the dispersed nanoparticles is represented in the figure by means of the red arrows: the MNPs work as nanoantennas in the amplification of the applied magnetic field.

A particularly interesting interpretation of the positive effect of an SEMF is rationalized in [108]. The authors evidenced that the applied external magnetic field is enhanced by the paramagnetic Fe_3_O_4_ nanostructures dispersed in the poly-glycolic acid hydrogel (scheme in Figure 3). This magnetic nano-field influenced the surficial receptors of the human umbilical-cord-derived mesenchymal stem cells seeded into the scaffold activating downstream signalling pathways. As a consequence, a significant improvement of gene expression of ALP, osteocalcin and type I collagen were measured. In vivo tests, performed on bone defects induced in rabbit radius, showed that the bone regeneration process was accelerated with an increase in bone mineral density, percentage of bone volume/tissue volume ratio and new bone area formation in the presence of MNPs and an external magnetic field. 

The in vivo bioactivity of bone scaffold based on CS/n-HA was further increased using lanthanum doping [75], even though this biological mechanism is not still completely clear. In particular, SrFe_12_O_9_ M-type hexagonal ferrite was used to give magneto-responsive behavior to lanthanum doped CS/n-HA (MNPs-La-CS/n-HA) scaffolds. The use of SrFe_12_O_9_ is justified since they are able to show a permanent magnetization after the application of static magnetic field during the scaffold formation. Morphological analysis was carried out by SEM. All the realized scaffolds showed a 3D structure with pores interconnection; furthermore, SEM pointed out that bone marrow mesenchymal stem cells (BMSCs) were intimately attached to the scaffolds. The live/dead cell staining evidenced a higher number of living BMSCs on MNPs-La-CS/n-HA than those ones observed on La-CS/n-HA and CS/n-HA. The influence of the effect of the magnetic nanoparticles and of the La doping on the cell polarization was evaluated by the incubation of pro-inflammatory macrophages (M2) and anti-inflammatory macrophages (M1). The experimental evidence showed that SrFe_12_O_9_ nanoparticles and La doping inhibited the differentiation of M1 and promoted the M2 macrophage polarization, then inducing a pro-bone repair environment. Finally, in vivo studies (rats calvarial defects), monitored by means of micro-CT [109], showed that the regeneration properties in the presence of MNPs-La-CS/n-HA appeared enhanced compared to La-CS/n-HA and CS/n-HA scaffold. Even the Yb dopant dispersed with SrFe_12_O_9_ in the CS/n-HA scaffolds promoted the vascularization and improved the osteogenic ability, showing promising features for enhancing osteogenic and angiogenic abilities bone defect repairing [110]. SrFe_12_O_9_ nanoparticles were used by the same authors, even in this case dispersed in the chitosan matrix, in the presence of porous bioglass microspheres (BG) with a diameter of about 300 nm [111]. CS powder was dissolved in acetic acid and the MNPs and BG were added. Samples were frozen at −20 °C for 12 h under external strong magnetic fields and then were freeze dried at −60 °C for 48 h. Two different ratios of SrFe_12_O_9_ and BG were used (1:7 and 1:3) and the scaffolds, both in the presence and in absence of MNPs appeared formed by a 3D macropores interconnected network. Undifferentiated human BMSCs cells were used for studying the cells’ behavior in the presence of the MNPs/BG/CS. In all tested scaffolds (BG/CS, MNPs/BG/CS 1:7 and MNPs/BG/CS 1:3), the hBMSCs were uniformly spread and they appeared non-toxic and a good cytocompatibility was observed. After the initial magnetization, the recorded magnetic field for the scaffold MNPs/BG/CS 1:7 and MNPs/BG/CS 1:3 is 0.21 and 0.52 mT, respectively. The presence of the magnetic field into the scaffolds promoted the hBMSCs‘ proliferation and the osteogenic differentiation higher in the case of MNPs/BG/CS 1:3, than for the MNPs/BG/CS 1:7 and for the bare BG/CS. Same conclusions were drawn on the in vivo experiments when the scaffolds were implanted in critical calvarial defect models and more bone tissues were promoted in both MNPs/BG/CS 1:7 and MNPs/BG/CS 1:3 than BG/CS scaffold. Furthermore, the authors evaluated the temperature increase obtained irradiating the scaffolds containing the MNPs by means of an NIR laser (808 nm, 4.6 W cm^−2^). The SrFe_12_O_19_ nanoparticles showed a broad absorption band centred at 900 nm (in the NIR region) so the 808 nm laser radiation is absorbed by the composite scaffold. This phenomenon induced a temperature increase in the bioglass (BG)/chitosan (CS) matrices enriched by the SrFe_12_O_19_ nanoparticles. With a prolonging irradiation time, a temperature increase up to about 46 °C is obtained after 6 min of laser irradiation. It is worth observing that temperatures higher than 41 °C induce the protein denaturation, so this approach could be used for the cancer cells treatment [112]. The efficacy of laser NIR irradiation of SrFe_12_O_19_ nanoparticles containing scaffolds for cancer treatment was monitored in vivo. There was not cancer cell apoptosis when the bioglass (BG)/chitosan (CS) scaffold was irradiated with the NIR source; on the contrary, tumor cell necrosis was observed when the in vivo experiments were carried out using the scaffold containing SrFe_12_O_19_ nanoparticles.

A composite layer of collagen and MNPs was deposited on a titanium plate by means of alternating potential-assisted electrochemical deposition [113]. Collagen I was dissolved in acetic acid and mixed with Ca(NO_3_)_2_ and (NH_4_)H_2_PO_4_ water solution. MNPs with ammine functionalization modified by polyethylene glycol (PEG) were added to the solution to form an electrolytic solution. The Ti plate was used as a cathode and a platinum plate as an anode in different experimental condition for immobilizing the collagen, MNPs, Ca^2+^ and PO_4_^3−^ ions on the cathode. With this approach, the authors were able to tune the MNPs position into the layer (into the bulk or close to the surface) [114]. A uniform distribution of MNPs on collagen fibers was observed in all cases and the shear strength of the scaffold with MNPs showed a higher shear strength. Both the simple presence of the MNPs and the application of an SEMF onto the scaffold enriched with the superparamagnetic nanoparticles enhanced the ALP activity for an MC3T3-E1 cell culture. In this case, the cellular morphology appeared dramatically influenced, changing from spindle fibroblast-like to polygonal shape.

Furthermore, the application of the magnetic field on the MNPs decorated mineralized collagen scaffold induced a better cell spreading, differentiation and osteogenesis-related gene expression in the MC3T3-E1 cells as a consequence of the deformation induced on the coating and on the intracellular magnetically actuated movement of MNPs.

As well as collagen, silk fibroin (SF) protein [115] was used to obtain a magneto-responsive scaffold in mixture with poly(2-hydroxyethyl methacrylate) and Fe_3_O_4_ MNPs [116]. Silk fibroin and poly(2-hydroxyethyl methacrylate) were synthesized by free radical polymerization technique in the presence of ethylene glycol dimethacrylate acting as cross linker. The MNPs were added to the slurry and the mixture was kept at 60 °C for 2 days and then dried at room temperature for 7 days. Mineralization of hydroxyapatite in scaffold matrix was obtained by an alternate soaking process: the samples were dipped in 50 mL CaCl_2_ water solution for 12 h at 37 °C and then in Na_2_HPO_4_ solution adjusted with HCl to reach pH 7.4 for 12 h at 37 °C; the procedure was repeated four times. The cytotoxicity of the scaffolds with and without the MNPs was tested on 3T3-E1 preosteoblasts by monitoring the LDH activity in the culture media. After 48 h in cell culture, a very interesting increase in LDH activity in the culture medium was observed and the cell viability and proliferation were not affected by the presence of MNPs. When a weak (120 mT) external static magnetic field is applied, the orientation of the cellular population along the magnetic field was forced. Furthermore, it is very fascinating to observe that the cellular proliferation potential appeared increased when the H is applied on the scaffolds without superparamagnetic nanoparticles and it was drastically enhanced when the scaffolds are enriched by the MNPs. As highlighted by the authors, the effect played by the magnetic field application on cell proliferation cannot be explained considering the single stimulus but the synergic effort of different triggers on the whole system. 

**Figure 4:**
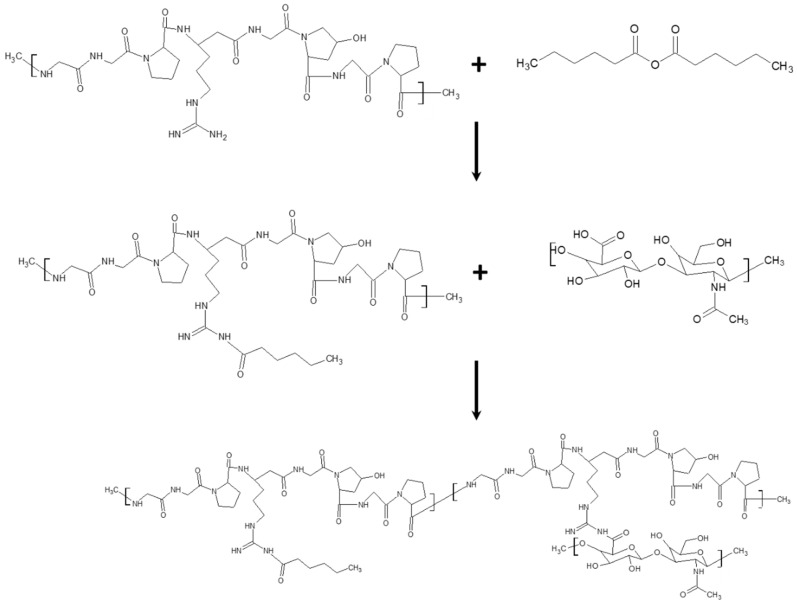
Synthesis route used in [117,118] to obtain the functionalized gelatine used for the magneto-responsive microcapsules.

Microcapsules composed by gelatine (extracted from porcine skin) and hyaluronic acid were realized according to a double emulsion process [117] (see Figure 4), and iron oxide MNPs were dispersed into the double layer of the microcapsules (MCs) forming a core-shell structure [118]. Rabbit primary chondrocytes and microcapsules were mixed and the CD44 receptors on the chondrocyte membranes attracted the MCs. The formed microcapsules in the presence and in absence of MNPs appeared not cytotoxic for cell culture even at a high MC concentration. Furthermore, sulphated glycosaminoglycan was evaluated as an index of the biochemical functionality of chondrocytes; its reduction suggests a de-differentiate tendency in fibrochondrocytes and physical stimulation was observed to enhance sulphated glycosaminoglycan secretion after 21 days. The in vivo tests were carried out by implanting a magnet in the head of the femur of a male New Zealand rabbit, which worked as magnetic field source, and the MCs were injected into the damaged cartilage. The simultaneous presence of MCs and magnetic stimulation improved the retention and bio-functionality of transplanted chondrocyte to form an ordered and structured arrangement in cartilage matrix. 

The potentiality of another gelatine-based scaffold for cartilage tissue engineering under pulse electromagnetic fields (of 100 mT) application was reported in [119]. Gelatine powder was dissolved with β-cyclodextrin in ultrapure water and γ-glycidoxypropyltrimethoxysilane as a cross-linker [120]. Superparamagnetic Fe_3_O_4_ nanoparticles were added to the solution and the mixture was stirred and the freeze dried for forming the hydrogel. Both the gelatine/β-cyclodextrin and gelatine/β-cyclodextrin/MNPs showed a porous structure even though the surface of the scaffold enriched with the MNPs appeared rougher than the gelatine/β-cyclodextrin scaffold improving the cell adhesion and proliferation. The compressive strength increased from 2.25 MPa up to 2.79 MPa when the MNPs were embedded in the hydrogel and the magnet field action significantly increased the expression of cartilage-specific gene markers for the case of bone marrow mesenchymal cells seeded on paramagnetic and gelatine/β-cyclodextrin/MNPs hydrogel. 

Even though a positive effect on the cells viability, proliferation and osteogenisis under the application of an external magnetic field on an opportunely fabricated scaffold was largely reported, many parameters have to be considered and monitored to induce relevant effects on the bio-activity. For example, as reported by Aldebs and co-workers, the application of a low-frequency pulsed electromagnetic fields on human adipose-derived mesenchymal stem cells seeded on a magneto-responsive hydrogel scaffolds showed a significant enhancement of the proliferation after 7 days of application [121]. On the other side, the beneficial effect of external pulsed magnetic field effect appeared reduced after 14 and 21 days of application. Probably, as suggested by the authors, this phenomenon was not due to the magnetic field but simply to the different number of MNPs in the scaffold and grown cells: MNP number remains unchanged but the cell number increased, reducing the beneficial effect of the magnetic nanoparticles. 

## 3. Magnetic Scaffolds for Soft Tissue Regeneration Applications

Soft tissues include a variety of tissues (muscular, cardiac, nervous, and skin tissue) characterized by low stiffness and high elasticity [122] and which have the function of supporting, surrounding and connecting the structures and organs of the human body [123]. Soft tissue injuries can be due to genetic defects, diseases, trauma and aging and can lead to a permanent impairment of their functions [124]. The restoring of the damaged soft tissue mechanical properties, in terms of elasticity, stiffness, elongation, is one of the main challenge of the soft engineering. These properties are key factors to guide cells adhesion, proliferation and differentiation during the regeneration process [125]. Recently, magnetic hydrogels have been gained more attention as biocomposites for soft tissue repair applications, thanks to the mechanical features and the sensitivity to an external stimulus, i.e., the magnetic field [27]. Magnetic scaffolds are indeed reported to induce tissue damages repair through diverse approaches depending on the kind of tissue to be repaired. For instance, it is possible to design anisotropic magnetic scaffold able to mimic the specific tissue microenvironment for the repair of soft tissue containing hierarchically organized structures [126]. Most soft biological tissues consist of an extracellular matrix that can be considered a fibrous polymer network containing highly organized nanocomposite structures. These ordered structures give the tissue essential anisotropic performance for important biological functions and for efficient cell–cell communication. The use of magnetic nanoparticles in tissue engineering allows one to easily create highly ordered structures within a hydrogel through the use of an external magnetic field [127]. Furthermore, MNPs embedded in a polymeric matrix are reported to be able to passively modulate the cellular response as their presence induces a specific substrate stiffness influencing the cellular behavior. In fact, their presence is connected to the improved mechanical integrity of the polymer in terms of localized reduction in deformation and increase in stiffness. The accumulation of these localized variations of the mechanical properties of the polymer induces an overall improvement in the stiffness of the entire matrix [128,129]. This technically means that the scaffold stiffness can be regulated by changing the embedded micro/nanoparticles amount [130], making the developed 3D structures more suitable to mimic a specific soft tissue rather than another one with different mechanical properties. Besides, the capability to stimulate the cellular response through the magnetic field makes these scaffolds even more interesting for the soft tissue regeneration. In fact, the highly organized cells of these tissues respond to topographical changes in the substrate or in general to mechanical stimuli through the activation/promotion of mechanotransduction pathways essential for tissue regeneration [131,132]. In particular, the mechanotransduction can be defined as the cells’ ability to respond to mechanical stimuli through biochemical signals [133]. By applying a magnetic field to magnetic NPs containing polymeric matrices, physical modifications close to the embedded MNPs can be induced creating heterogeneous forces that are perceived by the surrounding cells [129]. The magnetic stimulation of MNPs would therefore allow the mechanical regulation of different cellular functions such as the re-arrangement of the cytoskeleton and the alignment of the myotubes in muscle cells [29,134] or the regulation of intracellular calcium levels in excitable cells such as neurons and heart cells [29]. In the in vivo scenario, cell activity is coordinated by dynamic interactions with the extracellular matrix (ECM) through its space-time stiffening and softening, which is mediated by chemical or physical stimuli. Cells respond to mechanical changes of the ECM by constantly reshaping it [135,136]. A magnetic hydrogel can be designed to have reversible and tunable mechanical properties, such as viscoelastic properties [137] and load capacity (load bearing capacity) [138], in order to imitate the dynamic mechanical variations of the extracellular matrix improving the regeneration of many soft tissues [129]. For example, Abdeen and collaborators designed a magneto-active hydrogel based on polyacrylamide and carbonyl iron particles with elasticity and stiffness tunable upon external magnetic field application [139]. Such a hydrogel was characterized by a reversible possible modulation of the storage modulus (G’) between 0.1 and 80 kPa. Considering that G’ values reported for the soft tissues range from 0.1 to about 12–20 kPa [140,141] and that the obtained G’ value range was quite wide to cover most tissues in general, the proposed hydrogel can be thought for several applications in tissue engineering. Further, mesenchymal stem cells were grown on the developed scaffold and the spreading area, which is a key parameter to guarantee cell differentiation [142], was twice increased when the magnetic field was applied.

### Soft Tissue Cellular Response Modulation by Magnetic Scaffolds 

Among soft tissues, nervous tissue is one of the most studied as injuries can affect the quality of life of the patient causing long-term disability [143]. Mammalian neuronal cells have limited ability to re-grow and restore their functions after damage [144]. One of the main obstacles of neural regenerative medicine is the inability to restore the micropattern of the extra-cellular microenvironment avoiding the growth of scar tissue at the site of damage [145]. Central nervous tissue cells are surrounded by extracellular matrix (ECM) with a specific anisotropic architecture that supports tissue development and homeostasis [122]. Anisotropic polymeric implants characterized by the presence of aligned structures can provide the correct architecture to promote cell proliferation and directional nerve growth reducing scar formation at the site of the lesion during healing phases [146]. Injectable matrices are particularly suitable for supporting the healing of acute lesions in the central nervous system, such as spinal cord injury (SCI), having the advantage of being minimally invasive as they are injected directly in situ [147]. In this context, a hybrid injectable hydrogel called Anisogel was developed by Rose and collaborators to drive nerve growth and induce the regeneration of nervous tissue [148]. Anisogel has a dual nature based on human fibrin-based cross-linked hydrogel surrounding magnetic, soft, porous rod-shaped microgels. The microgel has a non-adhesive nature for cells instead of the fibrin network and this feature can drive anisotropic cells differentiation. Anisogel was characterized by a significant hierarchical geometry from the nano- up to the macro-scale which is fundamental for this kind of applications. Migrogels were synthesized with a mold-based soft lithography approach [149] using a reactive pre-polymer solicitation of star-shaped poly(ethylene oxide-stat-propylene oxide) with acrylate end groups (star-PEG-A) blended with different PEG-based diluents at different molecular weights in order to optimize the control on the induced mechano-physical properties of the microgels and thus the interaction with cells [150]. Anionic-coated MNPs were randomly dispersed in the pre-polymer solution before the molding procedure, allowing for the preferential orientation of the rodlike microgel axis parallel to the applied magnetic field in the mT range (130 mT). Further, the MNPs’ retention within Anisogel was investigated and confirmed in PBS 1X at 37 °C for 4 weeks. To study the biological functionality of the material, chicken-derived primary dorsal root ganglia (DRG) were seeded in the hydrogel during the cross-linking step inside a 130 mT magnetic field or without exposure to the magnetic field. Aligned microgels induced neurite-oriented growth alongside the microgel axis; randomly oriented microgels permitted instead random neurite growth. These results demonstrate how a 3D magnetic matrix with anisotropic characteristics can be used for the regeneration of sensitive and oriented tissues such as the nervous one. Anisogel fabrication was further improved [151] by using the electrospinning technique to ad hoc engineer microgels. Mixed solutions of fibers of poly (lactide-co-glycolide) (PLGA) and MNPs (average diameter, 5.2 ± 1.0 nm) were electrospun and then cut into short fibers (between 25 and 100 μm). The doped fibers were surrounded by a fibrin-based hydrogel inside a weak magnetic field (100 mT) during the cross-linking phase to align their main axis along the direction of the magnetic field lines. Primary neurons (L929, mouse-derived fibroblasts) and full embryonic chick DRG were incorporated within the 3D matrix; the elongation of fibroblasts and the unidirectional neurite growth along the direction of the fibers were confirmed. Moreover, PLGA fibers, if compared to PEG-based microgel, is characterized by good cell adhesion features improving cellular extension rate. In addition, the grown neurons in these conditions were able to recover their electrochemical activities; the electrical signals propagation took place in one direction, parallel to the fibers’ main axis. This type of 3D matrix has opened an important field of application of magnetic biomaterials that can be injected and used as cell guides in the regenerative processes of sensitive tissues. 

In this context, the electrospinning technique was also reported by Johnson and collaborators [152]. This fabrication technique is a powerful tool to generate highly aligned fibers that mimic the white matter tract characterized by the presence of long myelinated axons. In this case, the idea was to develop small injectable conduits based on pre-aligned electrospun fibers which could provide neurons directional growth and elongation. With this aim, poly-L-lactic acid (PLLA) fibers doped with different amounts of oleic acid coated MNPs have been electrospun according to a reported procedure [153] and coated with laminin in order to improve cell adhesion. These fibers were injected into collagen or fibrinogen solution, aligned by using static magnetic field (neodymium magnets) and used to provide directional guidance for neurite growth from ganglion explants (in 3D environment). Indeed, to assess the effect of fiber magnetization and topography on neurite growth, DRG were incorporated in both fibrin and collagen hydrogels containing PLLA fibers. The fibers were oriented using a magnetic field and the hydrogels were subsequently solidified to lock the fibers in the aligned configuration. A significant increase in neurite growth was observed in both hydrogels probably also due to the presence of oleic acid, which is considered a neurotrophic factor. In addition, neurite perceived the presence of fibers by remaining in contact with their surface and following their orientation during their growth. It was also observed that conduits generated by aligned fibers provided support for cellular growing, even when the hydrogel was degraded.

Another emerging and effective method for repairing and replacing damaged central nervous system tissues is based on the induction of exogenous stem cell neurogenesis [154]. In this context, a high-performance magneto-electric (ME) scaffold was proposed to induce neural cell differentiation without the use of chemical differentiation factors [155]. For the preparation of the scaffold, polyvinylidene difluoride (PVDF) in the crystalline β phase was obtained in order to exploit its piezoelectric features. Ferrite nanoparticles (CoFe_2_O_4_, CFO) of about 5 nm were used as magnetostrictive phase within the magneto-electrical scaffold (ME). In the case of ME composites, a magnetic field-induced motion in the magnetostrictive component is transferred to the piezoelectric component which converts the mechanical movement into polarization variations [156]. To avoid the agglomeration of ferrite nanoparticles, graphene oxide (GO) sheets were used to produce GO/CFO nanostructures. PVDF solutions containing GO/CFO have been electrospun to obtain fibrous scaffold [157]. Scaffold PVDF/GO/CFO showed higher electrical conductivity and lower elastic modulus values than matrices synthesized in the absence of GO/CFO structures. To study the scaffold ability to control cell growth and differentiation, mesenchymal stem cells (MSCs) were seeded and grown on 3D matrices for 21 days with and without magnetic stimulation. Cells were exposed 8 h/day, during these 21 days, with extremely low-frequency electromagnetic fields (1 mT; 50 Hz). By analyzing the obtained results of gene expression and immunocytochemistry assay studies, it was possible to underline that cells trended to differentiate into neuron-like cells when stimulated by the magnetic field, even in the absence of differentiation media. Indeed, magnetically stimulated cells grew aligned to the applied field. ME nanofibers (PVDF component) were also able to transduce the magnetic field exerted by the magnetostrictive part of the scaffold in a local electric charge affecting cellular behavior and alignment. The outstanding result that needs to be highlighted is that such a scaffold allows one to differentiate neural cells avoiding chemical differentiation factors which might be toxic for human health. 

After damage to the central nervous system, synaptic regeneration and neural circuit reconstruction play a crucial role in the effective recovery of the lesion. Tissue engineering and regenerative medicine are gaining more attention as approaches supporting the recovery of a lesion by cell repair and neuromodulation. Neuromodulation promotes neuroregeneration and neural repair by affecting signalling in the nervous system [158]. Magnetic hydrogels can be an innovative method to induce non-invasive neuromodulation, for example, by exploiting the sensitivity of ion channels to mechanical forces induced by magnetic fields. Magnetomechanical stimulations have been reported to modulate the opening and the closing of mechanosensitive ion channels in DRG neurons when cultured in 3D magnetic hydrogel based on hyaluronic acid (HA) [159]. The hydrogel was fabricated by mixing 4-arm-poly-ethylene glycol (4-arm-PEG) vinylsulfone with magnetic fluorescent microparticles (MMPs) bearing thiol groups with an average diameter of 1 µm and with HA-thiol, a high molecular weight polymer (700 kDa) typically present as the main component of brain/spinal cord ECM [160].

**Figure 5:**
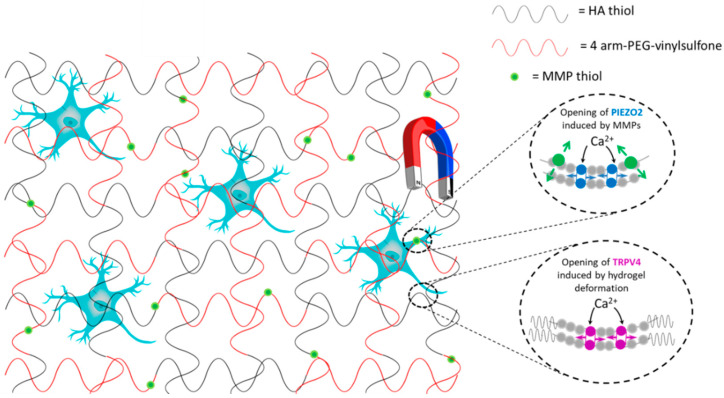
Schematic representation of the developed magnetic hydrogel based on 4-arm-PEG-vinylsulfone, HA and MMPs in [159] and of the magnetomechanical induced activation of Ca^2+^ channels: PIEZO2 and TRPV4. PIEZO2 channels are activated by the membrane stretching upon MMPs stimulations and TRPV4 channels were activated by the subsequent hydrogel deformation.

MMPs resulted homogenously dispersed within the HA hydrogel (5 μm apart from each other). Hydrogels such as these have biochemical and biophysical characteristics very similar to those of brain/spinal cord ECM and allow healthy growth of neurons. First of all, the 3D HA developed hydrogels were tested to check if primary DRG neurons could grow healthy. Then, the effect of acute magnetomechanical neural stimulation was investigated. During magnetic stimulation, Ca^2+^ 50% cellular influx increase was detected. The increase in Ca^2+^ influx was due to the opening of the endogenous mechanosensitive ion channels PIEZO2 and TRP4, activated by the mechanical stimulation. In detail, the magnetically activated microparticles were able to stretch cell membranes, and this activates PIEZO2. TRP4, instead, was activated by the deformation of HA chains under the magnetic field (Figure 5).

The modulation of various biological functions associated to signals mechanotransduction could be an interesting approach in the soft tissue engineering field for several reasons; among these reasons, thanks to deep tissue penetration feature of the magnetic field, this approach would permit in vitro and in vivo remote, non-invasive neural stimulation across deep brain tissues and potentially neurons in the peripheral nervous system [161] as well as of other kind of soft tissue characterized by the presence of mechanosensitive channels, as the cardiac one. 

As for peripheral nerves are concerned, these are fragile non-protected tissues that can be easily damaged by a variety of physical injuries. After damage, the peripheral nerve fails to recover its normal functions so nerve conduits are needed to build a regenerative tunnel [162]. In the last decade, engineered artificial nerves have been proposed as an alternative to autologous nerve grafting. Liu and collaborators proposed a magnetic scaffold based on chitosan-glycerophoshate and magnetic nanoparticles to improve the axonal regeneration of Schwann cells (SCs) and to recover the functionality of model rat sciatic nerve upon a defect of 15 mm [163]. The cylindrical structure with longitudinally oriented microchannels made the scaffold similar to the basal structure of a nerve and capable of driving neurite growth. The magnetic scaffold was demonstrated to have an appropriate rate of degradation, important for supporting nerve regeneration during the first 4–6 months, and was found to be biocompatible. Exposure of the magnetic scaffold to a magnetic field (MF, 2 mT, 50 Hz) improved the survival of the SCs in the first stage of the implant. In fact, after 12 weeks from the implant, the SCs, grown on the magnetic scaffold subjected to the magnetic field, were uniformly distributed; massive myelinated and regenerated axons were observed, spinal neurons and DRG sensory neurons were regenerated in the distal stump of the implant. The achieved result was comparable to the result obtained by using nerve autografts, employed as a control. Finally, but not less important, it has to be underlined that the motor function recovery of gastrocnemius muscles was obtained thanks to functional recovery of the sciatic nerve avoiding the muscle atrophy. 

Muscle is another soft defined tissue and tissue engineering strategies can provide alternative means to promote its repair and regeneration after damage. The functional capabilities of skeletal muscle tissue are strongly linked to its structure characterized by a highly ordered matrix of parallel fibers [164]. The maturation of muscle tissue in ordered structures increases the contractile forces of the myofibrils that lead to the generation of a total mono-axial force important for a correct skeletal muscle function [165]. Guiding the alignment of the multinucleated myotubes and inducing muscle maturity during the phases of tissue regeneration is a fundamental objective of skeletal muscle tissue engineering. In this context, a nano-composite hydrogel with anisotrope properties including MNPs within a methacryloyl gelatin matrix (Gelma) was developed [54]. The hydrogel has been subjected to the action of a weak magnetic field (20 mT) in order to allow for the self-assembly of the MNPs in oriented filaments. C2C12 cells (myoblast cell line) incorporated and grown in the 3D hydrogel are able to re-arrange their cytoskeleton to create long tubular structures aligned along the direction of MNP filaments. This morphological change leads to an early cellular maturation towards multinucleated myotubes without the use of differentiation media. Three-dimensional aligned paramagnetic anisotropic hydrogel could be a powerful tool to induce muscle tissue organization and cell morphogenesis during the regeneration of damaged and diseased tissues. Vannozzi and collaborators [166] designed a tubular scaffold to mimic the hierarchy of muscle tissue. In particular, self-folded tubular structures consisting of a bilayer of poly(ethylene glycol) diacrylate (PEGDA) were obtained. Both skeletal and cardiac muscle cells showed good viability. Further, magnetic nanoparticles of 50 nm diameter were incorporated in the hydrogel improving the modulation and the magnetic actuation properties of the system. Nonetheless, nowadays, the development of increasingly sophisticated actuators to mimic the hierarchy of native tissues in multiple scale is still a challenge. 

In addition to the shape and topography of the substrate, external stimulation such as electrical stimulation can promote muscle regeneration as it belongs to excitable tissues [167]. In a recent study, a promising hydrogel for muscle tissue regeneration has been fabricated [168]. A conductive ferrofluid was developed using a mussel-inspired approach and then used to prepare an anisotropic hydrogel that had both magnetic and conductive properties at the same time. The authors obtained magnetoeletric nanotubes through the polydopamine (PDA)-mediated in situ crystallization of Fe_3_O_4_ NPs on the surface of carbon nanotubes (PFeCNT) and subsequently dispersed them in an aqueous solution of PDA to obtain a ferrofluid. PFeCNT were obtained without the use of toxic substances and were found to be non-cytoxic as demonstrated by cell viability tests. PFeCNT dispersion was then incorporated in hydrogel containing acrylamide (AM) monomers in order to be aligned within the matrix under a magnetic field. AM moieties were polymerized according to an in situ free-radical polymerization leading to the fabrication of an anisotropic hydrogel with magnetic, conductive and self-adhesive characteristics. Moreover, the hydrogel could have improved mechanical properties depending on the amount and orientation of the nanotubes. In fact, when a tensile force was applied parallel to the alignment of the nanotubes, the “maximum extension ratio” increased compared to that obtained after the application of the same force in a perpendicular direction. The 3D polymer matrix also showed anisotropic behavior during compression tests. The effect of alignment, similar to that of muscle tissues, and the magneto-conductive properties of hydrogel on cellular behavior was evaluated by seeding C2C12 myoblasts. After only three days of incubation and under electrical stimulation, the cells proliferated and were elongated growing along the direction specified by PFeCNT nanohybrids. On the contrary, the cells grown on isotropic hydrogel, used as a control, showed no directionality. These results underline that the effects’ synergy, in particular, the structural and electrical anisotropy, can improve the cellular response. 

Mechanical stimulations are reported to strongly influence skeletal muscle tissue myogenesis; for example, mechanical stretching can induce myoblasts alignment, increasing myofibers diameter and improving muscle hypertrophy [169]. Direct mechanical stimulation can, therefore, improve the regeneration of many damages to skeletal muscle without the use of exogenous growth factors or cell therapies. A magnetic biphasic scaffold (Ferrogel) has been synthesized and implanted at the site of ischemic tibialis anterior muscle injury induced in mouse models to evaluate muscle regeneration following cycles of mechanical stimulation [33]. Ferrogel was based on alginate covalently linked with RGD peptide [33] and supplemented by 7 wt% of iron oxide nanoparticles. The injured muscle was subjected to compression stimuli at a frequency of 1 Hz for 5 min every 12 h for 14 days by approaching and retracting a permanent magnet. After 2 weeks of stimulation, each muscle area seemed to be active in regeneration and the sizes of the muscle fibers were larger (205 µm^2^) than those of the control (130 µm^2^), the ferrogel not stimulated. The cyclic mechanical stimulations led to a reduction in the thickness of the fibrous capsule after 2 weeks from the implant, from 120 µm in the control to 75 µm in ferrogel subjected to stress. In addition, it was observed how mechanical stimuli were able to significantly decrease fibrosis and inflammation of the diseased muscle; in fact, both M1 macrophages infiltration and interstitial fibrosis were reduced. Cyclic mechanical compressions were also able to stimulate the temporary increase in oxygen concentrations at the site of damage and to increase the specific tetanic strength peaks of the treated muscle. This study further assessed that mechanical stimulation can induce tissue regeneration and the magnetic scaffold can be a promising tool for modulating tissue responses without the use of bio-agents. Further, this is a technology that can be employed for other tissues opening an important scenario of applications.

Magnetic stimuli combined with magneto-responsive scaffold are even reported to achieve positive results in tendon tissue engineering [170]. Tendons have the function of transmitting to the bones the forces generated by the muscles; they are indeed exposed to high mechanical stress. To support large forces, the tendon has a highly organized structure consisting of type I collagen fibers arranged in dense parallel arrangements [33]. Tomás and collaborators proposed a 3D fibrous scaffold of poly-ε-caprolactone to mimic hierarchical architecture and biomechanical behavior of native tendons and to stimulate magneto-mechanically cellular responses [171]. To mechanically reinforce polymer fibers and to confer to them responsiveness to a magnetic field, rod-shaped hybrid cellulose nanocrystals decorated with superparamagnetic iron oxide (MNP@CNCs) were synthesized, according to a reported procedure of in situ precipitation [172] and incorporated in the scaffold. MNP@CNCs (216.5 ± 71.4 nm length and 4.3 ± 1.7 nm height) were coated with PDA and grafted with 1-dodecanethiol (DT-NP) to improve their polymer matrix compatibility, to make them more stable and to prevent aggregations in aqueous solutions. The polymer scaffold was synthesized by the electrospinning technique, the obtained PCL/DT-NP threads were then woven 12 times to obtain yarns mimicking the hierarchical fibrous architecture and the size of native tendon fascicles. To evaluate the biological performance of the system, the polymer scaffold containing 5 wt% of DT-NT was chosen, since it was found to have the highest mechanical properties and the highest magnetization of saturation (Ms) values while maintaining its own fibrous architecture. Human adipose stem cells (hASCs) have been seeded and grown on the constructs for 21 days with and without magnetomechanical stimulation. The magnetic conditions employed were: oscillation frequency of 2 Hz and 0.2 mm of horizontal displacement in a device consisting of an array of permanent magnets (arranged to fit under 24 well plates), each with a surface magnetic field of 0.35 T [173]. The magnetic field strength inside the wells was of around 0.30 T. No differences were found between the metabolic activity of cells magnetically stimulated and those in static cultures, suggesting that mechanical changes via magnetic actuation do not affect these cellular parameters. Concerning the morphology, stimulated cells exhibited improved mono-axial alignment and a more evident orientation along the longitudinal direction of the fibers than cells grown under static conditions. To assess whether the scaffold, upon magnetic stimulation (around 300 mT), was able to transport the mechanical stimulus to the cells seeded on it and then induce mechanisms of mechanoinjection, the authors followed the activation of YAP/TAZ shuttling in cells. This cytoplasmic protein dimer migrates into the nucleus activating genetic transcription only when mechanical stimuli, perceived by the cytoskeleton, are present. Cells magneto-mechanical stimulation was able to early activate YAP/TAZ triggering the transmission of the mechanical stimulus, which can be related to a higher cytoskeleton tension upon cell polarization. In addition, the magnetically stimulated cells expressed more SCX (Scleraxis) and TNMD (Tenomodulin) genes, which are considered the two largest tendon markers. These genes express crucial proteins for the induction of the tenogenic commitment of HASCs and for the regulation of the phenotype of tenocytes. Genetic expression analysis also showed that magnetic stimulation of the PLC/DT-NP scaffold could modulate the inflammatory response by preventing chronic inflammation and minimizing the formation of scar tissue when in vivo implanted.

**Figure 6:**
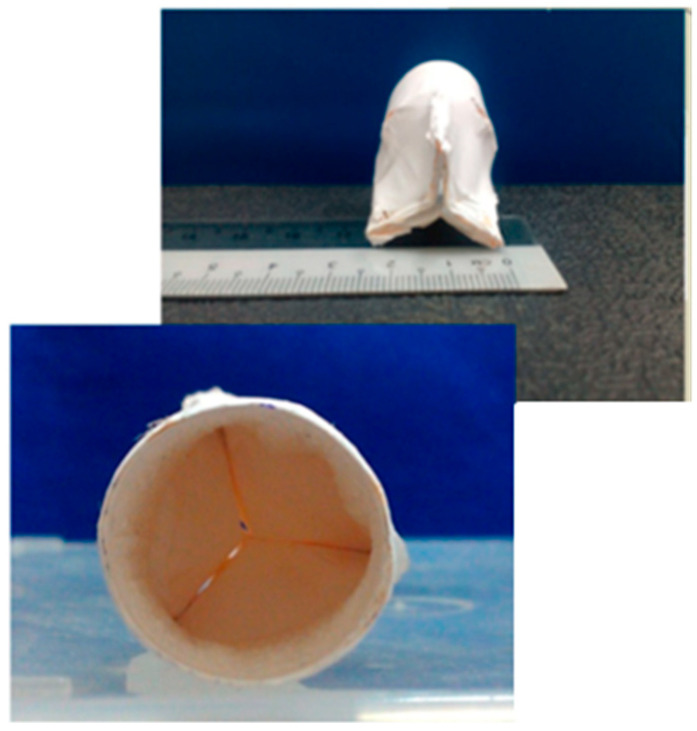
The 3D engineered aortic valve based on polyurethane/poly-L-lactic acid (TPU/PLLA) reported in reference [178]. In particular, 3.80% (*w*/*v*) MNPs were dissolved in TPU/PLLA mixture (50:50 *v*/*v*, 6.54 wt%); then, the mixture was subjected to electrospinning and collected into an aluminum foil based template of the valve obtaining the 3D scaffold reported.

The heart is one of the less regenerative organs of the adult mammal; in fact, cardiac cells cannot divide to replace injured cells [174]. Engineered magnetic scaffolds with adequate stiffness could support cardiac tissue regeneration by influencing the morphology of cardiac myocytes and the fate of stem cells [175]. Nazari and collaborators have developed a scaffold based on electrospun nanofibrous silk-fibroin (SF) containing 1% (*w*/*v*) of MNPs, to be used as a mechanical support for cardiac tissue regeneration [176]. MNPs were coated by a surface layer of casein to improve the dispersion within the SF matrix once incorporated with the aim to confer stiffness to the biological support. SF scaffold was obtained by an electrospinning approach starting from a mixture SF (11% *w*/*v*), polyethylene oxide (PEO, 5% *w*/*v*) and MNPs (1% *w*/*v*). The matrix was then desiccated for 6 h in the presence of ethanol vapour (75% *v*/*v*) in order to obtain SF β-sheets. The SF hydrogel tensile strength (MPa) and the final strain (%) increased as a result of the incorporation of MNPs, whilst the Young module decreased which means that the elasticity was slightly reduced, mechanical properties similar to those of the myocardium. Mouse embryonic cardiac cells (ECCs) seeded on SF scaffolds were positively affected by the fibrous topography and stiffness of the support improving the rate of survival and in general the proliferation. In addition, genetic and protein expression investigations showed how ECCs were able to differentiate towards adult heart cells (cardiomyocytes) confirming the key role played by the magnetic scaffold in terms of both cell proliferation and differentiation. Heart valve defects also need artificial support to be replaced. The heart has four valves which are responsible for directing blood flow from the body to the heart and vice versa [177]. The aortic valve works under a tensile-shear-flexural loading so it must have specific mechanical properties that will be different than the other valves. A 3D heart valve scaffold was fabricated [178] (Figure 6) based on polyurethane/poly-L-lactic acid (TPU/PLLA) and maghemite nanoparticles. Maghemite MNPs were obtained according to a coprecipitation procedure [179] and capped by citrate moieties to prevent oxidation and aggregation phenomena. A TPU/PLLA mixture (50:50 *v*/*v*, 6.54 wt%) containing 3.80% (*w*/*v*) MNPs was prepared, subjected to electrospinning and collected into an aluminum foil based template of the valve.

The electrospun scaffold was found to have a porosity of 90.72% and an adequate stiffness to support and improve the growth and proliferation of aortic smooth muscle cells during up to 34 days incubation. Macro-indentation tests were performed to follow the elastic modulus loss of the scaffold during cells incubation with promising results since, although the elastic modulus was reduced during time, the value was 50% larger than the required elastic modulus for the cardiac tissue.

**Figure 7:**
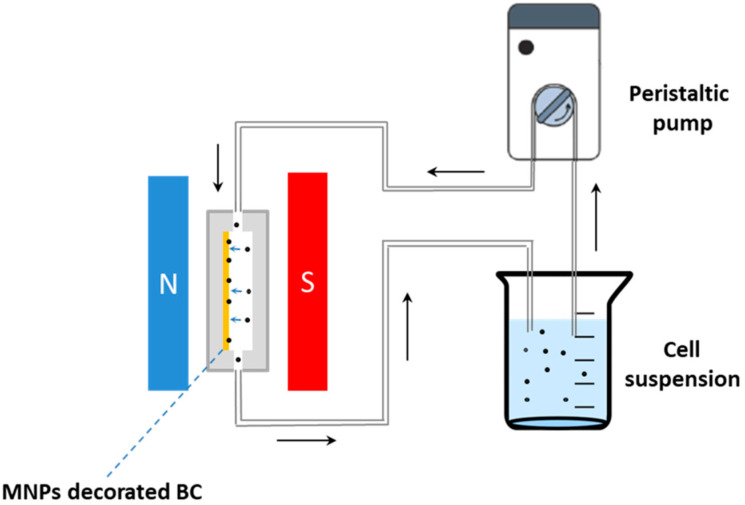
Schematic representation of the parallel-plate flow system used in [181]. Cells functionalized with MNPs were fluxed in the system under a magnetic field of 0.3 T and were magnetically attracted by the MNPs decorated bacterial cellulose (BC) hydrogel.

In the context of cardiovascular tissue, magnetic scaffolds were found to be very interesting for the repairing vascular defects. Endothelial dysfunction of a blood vessel can contribute to the evolution of various pathological conditions including aneurysms, stroke and heart disease, atherosclerosis. New approaches to repair these vascular defects are tissue-engineered vascular grafts (TEVG) which use derived-vascular cells with or without supports to allow endothelial repair of the vascular lesion [180]. The endothelial repair efficiency was, however, very low as about 95% cells are lost in the first 24 h from the implant due to the high hemodynamic stress. The localized cell targeting by magnetic means could be used to provide the strength required for cell retention so as to accelerate vascular remodeling. Arias and collaborators proposed a Fe_3_O_4_ nanoparticles embedding hydrogel based on bacterial cellulose (BC) to magnetically confine cells functionalized with magnetic nanoparticles and to release drug on demand at the site of vascular cell lesion [181]. MNPs incorporated in the hydrogel, when subjected to magnetic field, create a gradient of magnetic field that would locally direct the nanoparticles-loaded cells towards the surface of the hydrogel itself, so as to create a cell nest that would induce proliferation. Magnetite nanoparticles with a diameter of about 49.81 ± 20.77 µm were synthesized according to a coprecipitation approach [182] in situ at three different concentrations (25, 50 and 100 mM). From cytobiocompatibility studies performed using human aortic smooth muscle cells (HASMCs), it was observed that the magnetic hydrogel containing 25 mm of MNPs gave results comparable to the control, that is hydrogel without MNPs, and improved the rate of cell growth. In particular, MNPs used to decorate BC fibrils were coated with dextran moieties to avoid oxidation and to improve biocompatibility. Then, HASMCs were loaded with commercial dextran coated MNPs of 30 nm diameter upon 24 h of exposure. To verify that the magnetic hydrogel was able to attract magnetic HASMCs, a parallel-plate flow system was designed to magnetically attract on a BC support the circulating functionalized cells (Figure 7). When a magnetic field was applied (0.3 T), the MNP-loaded cells circulating in the flow chamber were captured on the surface of the magnetic scaffold creating a cell nest from which the proliferation of endothelial cells could start in vivo in order to obtain a fast and efficient endothelial cells proliferation leading to local vascular damage repair. 

In the repair of complex tissues such as heart or muscle, it is necessary to obtain pre-vascularized scaffolds in order to maintain cell viability during tissue growth, to induce structural organization, to promote vascularisation and integration with host tissue after implantation [183]. Magneto-mechanical stimulation is reported to be able to induce a direct response to the activities of endothelial cells and to influence their organization within 3D constructs [184]. The authors hypothesized that the exposure of a magnetic hydrogel to an alternating magnetic field could generate the right mechanical stimuli that would lead the endothelial cells to reorganize into early capillary-like structures in vitro. Magnetite-impregnated alginate scaffolds were synthesized by the freeze-drying technique. Morphological analysis shows how magnetite MNPs were homogeneously distributed within the matrix, making it more stiff and elastic than the control, non-loaded scaffold; however, its hydrogel-like behavior was preserved. The magnetic hydrogels and the non-magnetic ones were used to seed endothelial cells (ECs). Metabolic tests showed that the alternating magnetic field (frequency 40 Hz and intensity 1 mT) even in the case of the non-magnetic scaffold had a positive effect on cellular metabolic activity, which was further enhanced in the case of the magnetic matrix. After 14 days of incubation and exposure to the magnetic field, ECs grown on magnetic hydrogels were more organized in capillary loop-like structures than on the control scaffold where interconnected structures were observed almost lacking of loops. Probably, small deformations of the polymer matrix, given by the vibration of MNPs under magnetic field application, lead to a local magneto-mechanical effect on cells. As an alternative to this hypothesis, it is possible to suppose that the scaffold undergoes a general deformation under magnetic field, creating bending/stretching forces that exert a mechanical effect on the seeded cells. Several “bottom up” manufacturing systems have been used to attempt to build vascular-like structures from micro-scale toroidal cellular modules (micro-Tcms). Sun and collaborators, in this context, created microvascular-like structures through magnetic assembly [185]. Magnetic alginate microfibers (MAMs) were fabricated into a polydimethylsiloxane (PDMS) microfluidic device. Magnetite MNPs obtained by a coprecipitation procedure [186] were employed to confer magnetic features. Then, micro-rings were created by twisting the obtained fibers around an aluminum wire no more than 15 times. NIH/3T3 cells were seeded on the micro-rings of alginate and after 12 h they were able to self-assemble around the periphery of the alginate ring forming a stable circular layer. Micro-rings containing 24 h grown cells were micro-stacked by contactless magnetic micro-assembly: an electromagnetic needle was used to move micro-rings inside a dish, filled by an aqueous solution, in order to direct and stack them in a copper micro-Pillar. A maximum of 19 micro-rings were axially assembled. After cells re-culturing for 12 h, the microrings began to adhere to each other as the cells clumped together, thus becoming a single microvascular-like structure, demonstrating the feasibility of the system as a scaffold for vascular tissue engineering.

Further, anisotropic magnetic scaffold based on collagen was recently reported to influence skin cells [187]. The author synthesized silica rods functionalized with iron oxide nanoparticles (SiO_2_@Fe_3_O_4_) and incorporated them into a type I collagen-based hydrogel. Silica rods were synthetized by a typical sol-gel procedure properly modified [188] and then decorated with 10 nm diameter MNPs obtained by a polyoil approach [189]. SiO_2_@Fe_3_O_4_ were mixed with the acidic solution of type I collagen to obtain the scaffold by a pH-controlled self-assembly approach [190]. The hydrogel was then subjected to a magnetic field by placing it between two parallel plate magnets (50–80 mT) and, in 5 min, structures aligned parallel or perpendicular to the hydrogel surface were obtained depending on the magnetic field lines’ orientation. Three kinds of samples were checked in terms of Normal Human Dermal Fibroblast (NHDF) cell growth and orientation: scaffold with parallel oriented structures, with perpendicular oriented structures and with no oriented structures. Since collagen represents a much more favourable surface for cell seeding if compared to SiO_2_ surface, NHDF cells preferentially grew in the collagen-rich areas avoiding silica rods, that, when oriented, represented proper growth guides in the magnetic induced direction. In particular, parallel oriented rods were able to create well-defined channels for cells growing, whilst the perpendicular oriented ones left more space to cells. These results represent a very promising approach for the realization of magneto-responsive matrices able to create cell growing guidance pathways, fundamental for regeneration of diverse soft tissue cells, including skin cells. 

## 4. Conclusions

The effect of the paramagnetic and superparamagnetic nanoparticles in bio-scaffolds for tissue regeneration has been considered. The huge number of contributions in this research field prompted us to separate the studies about supports fabricated for hard tissue application from the papers related to the regeneration of soft tissues. For both the macro-topics, solid scaffolds and hydrogels were considered, paying particular attention to the magneto-mechanical properties tuned by the presence of the magnetic nanoparticles. We can recognize that iron oxide nanoparticles were almost exclusively used as magneto-responsive nanostructures for their bio-compatibility and the easiness to synthetize them with different shape, size and functionalization. Most of the contributions demonstrated that the presence of MNPs enhance the mechanical properties of the scaffold where they are dispersed and, depending on their concentration, contribute to promote cell proliferation and differentiation. In fact, if analysed at the nanoscale, the superparamagnetic nanoparticles exert a magnetic field that influence the cell behavior promoting the proliferation and the differentiation. This aspect is often enhanced by the application of an external (static and/or oscillating) magnetic field that acts as a mechanical stimulus too. In fact, the scaffold mechanical stress can be transferred to seeded cells promoting the activation of specific bio-pathways. Several in vivo applications confirm the positive effect of the MNPs, their biocompatibility and suggest this solution as a very promising approach for the tissues’ regeneration and repair.
